# *Pareiorhina hyptiorhachis*, a new catfish species from Rio Paraíba do Sul basin, southeastern Brazil (Siluriformes, Loricariidae)

**DOI:** 10.3897/zookeys.315.5307

**Published:** 2013-07-04

**Authors:** Gabriel de Souza da Costa e Silva, Fábio Fernandes Roxo, Claudio Oliveira

**Affiliations:** 1 Laboratório de Biologia e Genética de Peixes, Departamento de Morfologia, IB-UNESP, Campus de Botucatu, 18618-000, Botucatu, SP, Brazil

**Keywords:** Cascudinhos, Taxonomy, Freshwater, Neoplecostominae, Neotropical Region

## Abstract

*Pareiorhina hyptiorhachis* is described from Ribeirão Fernandes and Rio Pomba, Rio Paraíba do Sul basin, Brazil. The new species is distinguished from its congeners (*Pareiorhina brachyrhyncha*, *Pareiorhina carrancas*, *Pareiorhina cepta*, and *Pareiorhina rudolphi*) by the presence of a conspicuous ridge on the trunk posterior to the dorsal fin (postdorsal ridge), simple teeth, a completely naked abdomen, a round dorsal profile of the head, greater suborbital depth and greater head width. We discuss the distributional pattern of the new species and its congeners and hypothesize that headwater capture is responsible for the distribution of *Pareiorhina* species across different watersheds in southeastern of Brazil.

## Abstract (pt)

*Pareiorhina hyptiorhachis* é descrita do ribeirão Fernandes e do rio Pomba, bacia do rio Paraíba do Sul, Brasil. A nova espécie é distinguida de suas congêneres (*Pareiorhina brachyrhyncha*, *Pareiorhina carrancas*, *Pareiorhina cepta*, e *Pareiorhina rudolphi*) por caracteres relacionados a presença de uma quilha conspícua na superficie pós-dorsal do tronco, dentes simples, abdomen completamente nú, perfil dorsal da cabeça arredondado, altura do sub-orbital e largura da cabeça. Nós discutido o padrão de distribuição da nova espécie e de seus congêneres e a hipótese de que a captura de cabeceira é responsável pela distribuição das espécies de *Pareiorhina* através das diferentes bacias hidrográficas do sudeste do Brasil.

## Introduction

The genus *Pareiorhina* was proposed by Gosline (1947) to include *Rhinelepis rudolphi* Miranda-Ribeiro, 1911 and is currently included in the subfamily Neoplecostominae (sensu Chiachio et al. 2008; Roxo et al. 2012a, 2012b). Recently, three new species have been described: *Pareiorhina carrancas* by Bockmann and Ribeiro (2003); *Pareiorhina brachyrhyncha* by Chamon et al. (2005); and *Pareiorhina cepta* by Roxo et al. (2012c). In their description of *Pareiorhina carrancas*, Bockmann and Ribeiro (2003) proposed a combination of characters to separate *Pareiorhina* from other genera of Loricariidae: the lateral borders of the head lacking hypertrophied odontodes; unicuspid teeth; a naked abdomen; dorsal plates meeting along the mid-dorsal line between the dorsal and caudal fins; adipose fin absent; ventral plates covering the mid-ventral line behind the anal-fin base; and the dorsal portion of the body behind the dorsal fin flattened. However, no exclusive synapomorphies were presented to diagnose the genus. Recently, the molecular studies of Cramer et al. (2011) and Roxo et al. (2012a, 2012b) have found that *Pareiorhina* may not be monophyletic.

An examination of the fish collections at the Laboratório de Biologia e Genética de Peixes de Botucatu (LBP) and Museu de Ciências e Tecnologia, Pontifícia Universidade Católica do Rio Grande do Sul (MCP) revealed the existence of an undescribed *Pareiorhina* species from the Rio Paraíba do Sul basin, Brazil. This new species is formally described herein.

## Material and methods

All measurements were taken from point to point to the nearest 0.1 mm using digital calipers (except the postdorsal ridge depth, which was measured using a stereomicroscope and analyzed using the software Axio Vision Release 4.8.2). Counts were taken from the left side when possible. In the description, counts are followed by their frequencies in parentheses. The measurements followed Bockman and Ribeiro (2003), except for the folded dorsal-fin length and the snout-opercle length that were not included in that publication. We added the following measurements from Carvalho and Reis (2009): mandibular ramus, suborbital depth and unbranched anal-fin ray length. We also added the measurement of postdorsal ridge depth (from the base of the postdorsal ridge to its upper portion). Osteology was performed on specimens cleared and double-stained (c&s) according to the procedures of Taylor and Van Dyke (1985). The osteological and the body-plate nomenclature followed Schaefer (1997). Vertebral counts were obtained from cleared-and-stained specimens and included the first five vertebrae modified into the Weberian apparatus. The compound caudal centrum (PU1 + U1; Lundberg and Baskin 1969) was counted as one vertebra. The pores nomenclature followed Arratia and Huaquin (1995). Asterisks in the text refer to the holotype. After collection the animals were anesthetized using 1% benzocaine in water and fixed in 10% formalin for at least two days, then transferred to 70% ethanol for permanent storage for morphological studies.

All examined material was housed at the following Brazilian institutions: LBP (Laboratório de Biologia e Genética de Peixes, Universidade Estadual Paulista Júlio de Mesquita Filho, Botucatu - SP); MCP (Museu de Ciências e Tecnologia, Pontifícia Universidade Católica do Rio Grande do Sul, Porto Alegre - RS); MZUSP (Museu de Zoologia da Universidade de São Paulo, São Paulo - SP); and NUP (Coleção Ictiológica do Núcleo de Pesquisas em Limnologia, Ictiologia e Aquicultura, Universidade Estadual de Maringá, Maringá - PR).

## Results

### Pareiorhina hyptiorhachis sp. n.

urn:lsid:zoobank.org:act:1D6D4D43-68CF-485B-9ABC-8FFB270E2460

http://species-id.net/wiki/Pareiorhina_hyptiorhachis

Figure 1 ; Table 1

Pareiorhina sp. 1 - Roxo et al. 2012a : 2443 [phylogenetic relationships]. - Roxo et al. 2012b: 38 [phylogenetic relationships].

#### Holotype.

MZUSP 111956, female, 33.6 mm SL, Brazil, Minas Gerais State, municipality of Santa Bárbara do Tugúrio, Ribeirão Fernandes, a tributary of Rio Pomba, Rio Paraíba do Sul basin, 21°14'47"S, 43°34'07"W, 19 Jun 2011, Ferreira AT, Roxo FF, Silva GSC.

#### Paratypes.

Brazil, Minas Gerais State, municipality of Santa Bárbara do Tugúrio, Rio Paraíba do Sul basin. LBP 12248, 2 males, 4 females, 1 c&s, 26.6–34.8 mm SL, collected with holotype. NUP 14331, 1 female, 29.6 mm SL, collected with holotype. LBP 1093, 1 male, 33.4 mm SL, Ribeirão Fernandes, 21°14'47"S, 43°34'07"W, 12 Oct 2001, Oliveira JC, Alves AL, Sato LR. LBP 8368, 5 females, 27.9–34.4 mm SL, Rio Pomba, 21°14'07"S, 43°30'50"W, 19 May 2009, Oliveira C, Silva GJC, Roxo FF, Pereira TNA. LBP 12257, 1 female, 27.2 mm SL, Rio Pomba, 21°14'07"S, 43°30'50"W, 19 Jun 2011, Ferreira AT, Roxo FF, Silva GSC. MCP 29432, 3 male, 1 female, 2 unsexed, (1 juvenile not measured) 23.8–39.0 mm SL, Ribeirão Fernandes, 21°14'47"S, 43°34'07"W, 12 Aug 2001, Oliveira JC, Alves AL, Sato LR.

#### Diagnosis.

*Pareiorhina hyptiorhachis* is distinguished from its congeners, except for *Pareiorhina carrancas*, by the presence of a postdorsal ridge (vs. the absence of a postdorsal ridge). The new species differs from *Pareiorhina carrancas* by having a more elevated postdorsal ridge, (Fig. 2; 16.7–26.8% of CP depth vs. 4.47–9.03%; table 1). Additionally, the new species can be distinguished from *Pareiorhina cepta* by having a naked abdomen (vs. having small plates covered with odontodes irregularly distributed on the abdomen); from *Pareiorhina brachyrhyncha* and *Pareiorhina cepta* by having unicuspid teeth (vs. teeth with a minute lateral cusp); from *Pareiorhina carrancas* and *Pareiorhina rudolphi* by having the anterior profile of the head rounded in dorsal view (vs. elliptical; Fig. 3) and by having a greater suborbital depth (35.0–40.5% of HL vs. 27.4–34.2% in *Pareiorhina carrancas* and 24.5–31.8% in *Pareiorhina rudolphi*). Moreover *Pareiorhina hyptiorhachis* is distinguished from its congeners by having a wider head (100.1–108.6% of HL vs. 91.7–98.1% in *Pareiorhina brachyrhyncha*, 82.9–96.2% in *Pareiorhina carrancas*, 83.4–90.5% in *Pareiorhina cepta* and 77.8–82.1% in *Pareiorhina rudolphi*).

**Figure 1.**
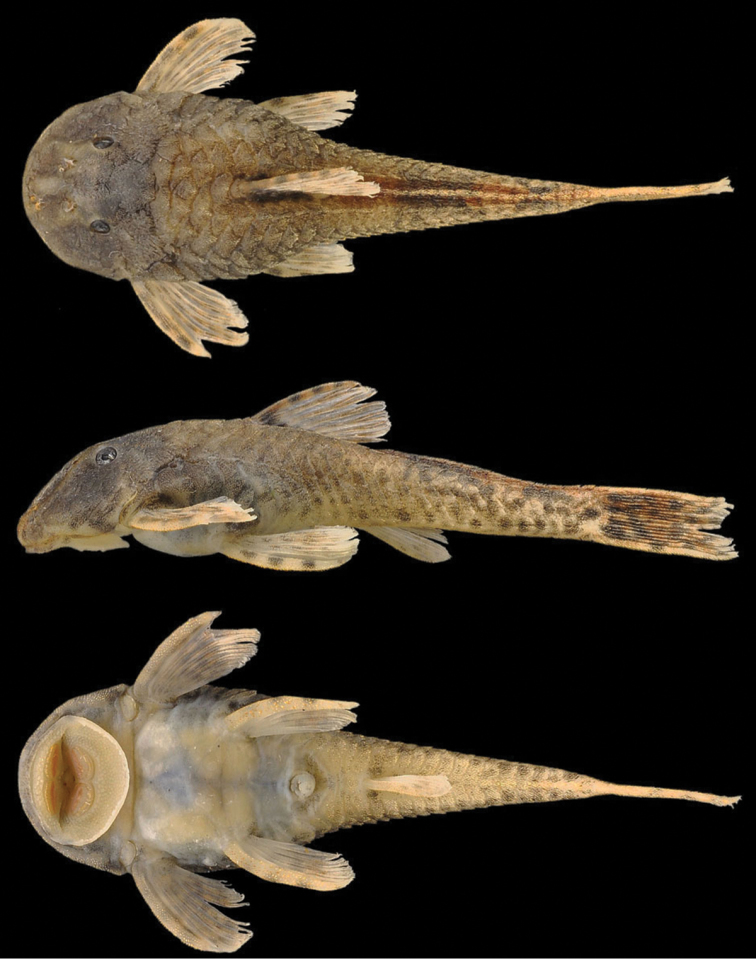
*Pareiorhina hyptiorhachis*, sp. n., MZUSP 111956, 33.6 mm SL, holotype from Ribeirão Fernandes, Rio Paraíba do Sul basin, municipality of Santa Barbara do Tugúrio.

**Figure 2.**
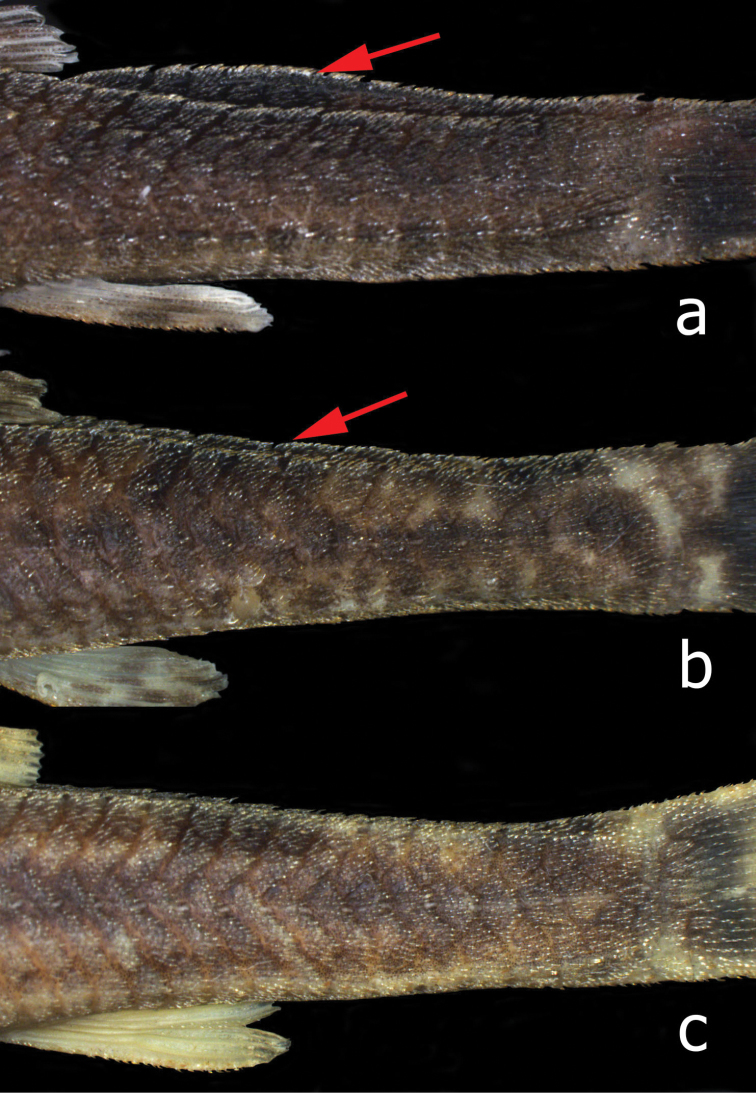
**a** *Pareiorhina hyptiorhachis*, sp. n.,paratype, LBP 12248, 29.2 mm SL, showing the conspicuous postdorsal ridge **b** *Pareiorhina carrancas*, LBP 8380, 38.2 mm SL, showing the poorly-developed postdorsal ridge **c** *Pareiorhina rudolphi*, LBP 8044, 40.5 mm SL, showing the absence of a postdorsal ridge.

**Figure 3.**
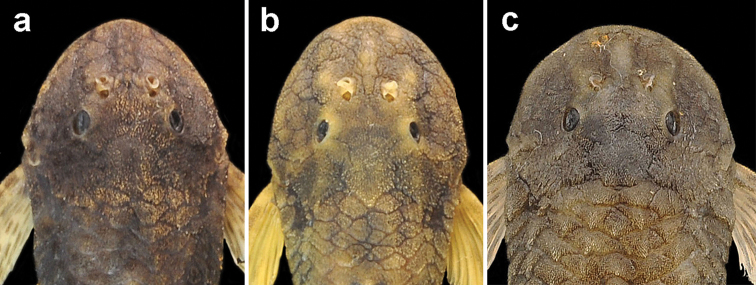
**a** *Pareiorhina carrancas*, LBP 8380, 36.5 mm SL, showing the elliptical anterior profile of the head elliptical in dorsal view **b** *Pareiorhina rudolphi*, LBP 8044, 42.0 mm SL, showing the elliptical anterior profile of the head in dorsal view **c** *Pareiorhina hyptiorhachis*, new species, holotype, MZUSP 111956, 33.6 mm SL, showing the rounded anterior profile of the head in dorsal view.

#### Description.

Morphometric and meristic data are given in Table 1. In lateral view, dorsal profile of body strongly convex from snout tip to distal margin of supraoccipital; straight from supraoccipital to dorsal-fin origin; slightly decreasing to end of caudal peduncle. Ventral surface of body, slightly concave at head portion, straight to convex from posterior end of head to pelvic-fin insertion, and straight but angled to posterior end of caudal peduncle. Snout tip rounded in dorsal view. Nostril small. Trunk and caudal peduncle rectangular in cross-section.

**Table 1.** Morphometric data for *Pareiorhina hyptiorhachis*. SD = Standard Deviation, IO = Interorbital, OD = Orbital Diameter, CP = Caudal Peduncle.<br/>

	***Pareiorhina hyptiorhachis* n=21**			
	**Holotype**	**Range**	**Mean**	**SD**
**Standard length (SL)**	33.6	26.6–38.8	31.0	3.0
**Percents of Standard length (SL)**				
Predorsal length	44.2	41.5–48.8	44.7	1.6
Preanal length	59.2	56.1–65.9	60.5	2.4
Head length	31.7	28.6–35.5	31.8	1.5
Cleithral width	32.8	30.4–36.9	33.5	1.8
Dorsal-fin unbranched ray length	21.2	20.3–24.1	22.2	1.1
Base of dorsal fin length	15.4	14.3–18.3	16.5	1.1
Thorax length	18.1	15.1–19.6	17.0	1.3
Pectoral-fin unbranched ray length	20.5	20.5–26.0	22.6	1.5
Abdomen length	27.0	22.6–30.1	26.2	1.6
Pelvic-fin unbranched ray length	22.5	17.7–26.6	22.9	2.1
Anal-fin length	15.3	13.7–17.8	15.6	0.9
Ventral unbranched caudal ray	24.9	20.3–30.5	25.2	2.8
Caudal-peduncle depth	9.0	8.3–11.0	9.39	0.7
Postanal length	34.9	31.6–38.1	33.9	1.4
Anal width	15.4	11.3–16.0	14.0	1.5
**Percents of Head Length (HL)**				
Head width	103.8	100.1–108.6	103.8	2.6
Head depth	61.7	53.5–62.8	56.9	2.3
Snout length	63.1	58.0–64.7	61.2	1.9
Interorbital width	37.7	34.8–40.7	38.0	1.4
Orbital diameter	11.4	11.1–15.5	12.7	1.6
Suborbital depth	39.2	35.0–40.5	37.3	1.6
Mandibular ramus	18.1	16.0–23.4	19.9	1.9
**Other measurements (expressed as percentages)**				
Anal width/cleithral width	47.1	32.0–49.7	42.0	5.1
IO/OD	29.5	21.6–42.1	33.5	4.5
IO/Mandibulary ramus	50.6	44.1–62.5	52.4	5.6
Predorsal length/first ds ray length	47.9	45.7–54.5	49.7	2.6
Postanal length/CP depth	25.8	24.8–31.1	27.6	1.9
Pelvic-fin length/CP depth	40.0	36.0–46.8	41.1	3.0
Ventral unbranched caudal ray/CP depth	36.2	32.1–46.0	37.5	4.3
Postdorsal ridge depth/CP depth	19.0	16.7–26.8	21.5	3.4

Greatest body depth at dorsal-fin origin. Body progressively narrowing posteriorly from cleithrum. Head flat to slightly convex between orbits; superior margin of orbits elevated. Eye small, orbital diameter 11.1–15.5% of HL, situated dorsolaterally just posterior of midpoint of head. Rostral margin of snout with minute, posteriorly-directed odontodes; numerous small odontodes on dorsal portion of head. Opening of swimbladder capsule small. Perforations of compound pterotic distributed on whole bone, greater and more concentrated on its ventral margin; its openings nearly rounded in median region, and irregular along inferior and posterior margins of bone. Lips large; oral disk rounded, papillose; premaxillary teeth 22 (1), 29 (1), 30 (1), 32 (1), 33 (1), 34 (2), 36 (1)*, 37 (2), 38 (1), 39 (2), 40 (2), 42 (2) or 44 (1). Dentary teeth 17 (1), 21 (1), 23 (1), 28 (1), 30 (2), 32 (2)*, 33 (2), 34 (2), 35 (1), 36 (1), 39 (1), or 45 (1). Teeth unicuspid. Maxillary barbel short and free distally.

Dorsal-fin rays ii,7; dorsal-fin originating at vertical through posterior end of pelvic-fin base; distal margin slightly convex. Pectoral-fin rays i,6; distal margin slightly convex; unbranched pectoral-fin ray reaching middle of unbranched pelvic-fin ray; unbranched pectoral-fin ray covered with large and pointed odontodes. Pelvic-fin rays i,5; distal margin of fin slightly convex; tip of adpressed pelvic fin almost reaching anal-fin origin; unbranched pelvic-fin ray covered with conspicuously pointed and well-developed, and uniformly distributed odontodes which are larger at ventral portion. Anal-fin rays i,5; distal margin slightly convex. Caudal fin rays i,7-7,i. Adipose fin absent. Caudal fin truncated with ventral unbranched principal ray longer than dorsal ray.

Body entirely covered by bony plates, except for ventral surface of head, abdomen and region overlaying swimbladder capsule. Dorsal series of plates 24–26, mid-dorsal 17–21, median perforated plates 24–26, mid-ventral 17–22, and ventral 19–22. Trunk with conspicuous, elongated, postdorsal ridge formed by 13–15 raised, unpaired, median plates; ridge continuous posteriorly with procurrent caudal-fin rays. Head lacking crest. Head and body plates covered with minute, uniformly sized and distributed odontodes. Seven pairs of ribs associated with vertebrae 8–14. Ribs slender and poorly ossified. Total vertebrae 29.

Supraorbital sensory canal with four pores; pore s1 located on prenasal plate below nasal plate; pore s3 located on posterior portion of nasal; pore s6+s6 located between frontal plates, on horizontal line through anterior limits of eye; pore s8 located on division between frontals, sphenotic and supraoccipital plates, just above eye. Infraorbital sensory canals with six pores; pore io1 located on anterior portion of first infraorbital; pore io2 located in medial region between first and second infraorbitals; pore io3 located in medial region between second and third infraorbitals; pore io4 located in medial region between third and fourth infraorbitals; pore io5 located in medial region between fourth and fifth infraorbitals and pore io6 located between sixth infraorbital and sphenotic. Preopercular canal with three pores; pore pm2 located on ventral portion of cheek plate, pore pm3 located between cheek plate and preopercle; pore pm4 located between preopercle and compound pterotic. Two postotic pores; pore po2 located just above of branchial slit; pore po3 located in region of overlying opening of swim-bladder capsule.

#### Color in alcohol.

**Figure 4.**
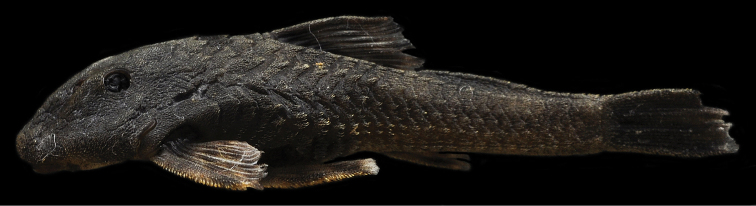
Additional coloration pattern of coloration of *Pareiorhina hyptiorhachis*, sp. n., LBP 12257, female, 27.2 mm SL.

Two body-coloration patterns observed. First pattern (Fig. 1): Ground color of dorsal surface of head and body yellowish brown. Ventral surface of body and head lighter than dorsal with dark spots of melanophores widely separated. Three dark saddles on dorsal surface of trunk (in some specimens not present), most anterior one inconspicuous. Lateral portion of body with inconspicuous dark stripe from head to caudal fin. Pectoral, pelvic and dorsal fins with three irregular, poorly defined bands. Caudal fin with variegated blotches. Second pattern (Fig. 4): Ground color of body uniformly dark except, ventral portion of body mostly clear; Fins with inconspicuous, irregularly defined bands: one in anal fin, two in pectoral and pelvic fins. Dorsal and caudal fins entirely dark.

#### Sexual dimorphism.

Males with a papilla at urogenital opening and fewer teeth in premaxillary 22–39 (*vs*. 32–44 females) and 17–32 dentary (*vs*. 30–45 females).

#### Etymology.

The specific name, *hyptiorhachis* is a combination of Greek, *hyptios* = supine, lying on the back, and *rhachis* = ridge, midrib, and is in reference to the conspicuous postdorsal ridge found in this species.

#### Distribution and habitat.

**Figure 5.**
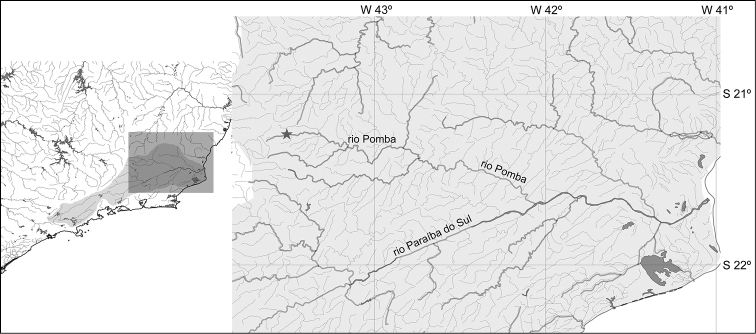
The Rio Paraíba do Sul basin indicating the type locality of *Pareiorhina hyptiorhachis* in Ribeirão Fernandes, a tributary of Rio Pomba, Rio Paraíba do Sul basin, 21°14'47"S, 43°34'07"W.

*Pareiorhina hyptiorhachis* is known from Rio Pomba and one of its tributaries, the Ribeirão Fernandes, in the municipality of Santa Barbara do Tugúrio, Minas Gerais State, Brazil (Fig. 5). This species inhabits moderate to fast-flowing streams, with a substrate of rocks and sand and margins covered by aquatic vegetation. Specimens were collected in association with loose stones, on the streambed. The new species is syntopic throughout its distribution with *Astyanax* sp., *Characidium* sp., *Geophagus brasiliensis*, *Harttia* cf. *carvalhoi*, *Imparfinis* sp., *Neoplecostomus microps*, *Trichomycterus* cf. *alternatus*, and *Trichomycterus* sp.

## Discussion

Bockmann and Ribeiro (2003) proposed seven characters to diagnose *Pareiorhina*. The new species described herein, *Pareiorhina hyptiorhachis*, possesses all of these characters. On the other hand, *Pareiorhina* did not form a monophyletic group in the molecular analysis of Roxo et al. (2012a, 2012b); in that analysis, *Pareiorhina hyptiorhachis*, cited as *Pareiorhina* sp. 1, appeared as the sister group of *Pareiorhina carrancas*, and these two species formed the sister group of *Neoplecostomus*. Furthermore, *Pareiorhina rudolphi*, the type species of *Pareiorhina* was the sister group of *Pseudotocinclus*. Considering that *Pareiorhina hyptiorhachis* exhibits all of the characters listed by Bockmann and Ribeiro (2003) for *Pareiorhina*, the molecular data conflict with the available morphological data for Neoplecostominae, and new morphological studies in Neoplecostominae are in progress (Edson Henrique Lopes Pereira, pers. comm.), we prefer to include *Pareiorhina hyptiorhachis* in *Pareiorhina* rather than in another Neoplecostominae genus or in a new genus.

*Pareiorhina hyptiorhachis* is similar to *Pareiorhina carrancas* from the upper Rio Paraná basin. The two species share unicuspid teeth and the presence of a postdorsal ridge of unpaired plates, although the postdorsal ridge is better developed in *Pareiorhina hyptiorhachis* (all female and male samples) (Fig. 2). Moreover, the new species has more raised median unpaired plates in the postdorsal ridge (13–15 vs. 10–13 in *Pareiorhina carrancas*). The close relationship between *Pareiorhina hyptiorhachis* and *Pareiorhina carrancas* suggested by the molecular data of Roxo et al. (2012b) is thus at least superficially supported by morphology.

*Pareiorhina* is distributed across three hydrographic basins, with *Pareiorhina rudolphi*, *Pareiorhina brachyrhyncha* and *Pareiorhina hyptiorhachis* from the Rio Paraíba do Sul basin; *Pareiorhina carrancas* from the upper Rio Paraná basin; and *Pareiorhina cepta* from the Rio São Francisco basin. Ribeiro et al. (2006) suggested that the activation of old faults in southeastern Brazil during the Miocene and Pliocene resulted in several headwater captures between adjacent drainages of the São Francisco, upper Paraná and Coastal rivers. Roxo et al. (2012a) suggested that the lineage that gave rise to *Pareiorhina carrancas* and *Pareiorhina hyptiorhachis* was from the upper Rio Paraná basin and that *Pareiorhina hyptiorhachis* reached the Rio Paraíba do Sul basin about 6.2 (2.3–11.2) million years ago, probably through headwater captures between the upper Paraná and several coastal drainages (Rio Paraíba do Sul and Ribeira do Iguape basin) during the late Miocene. Chamon et al. (2005) suggested that the evolutionary history of *Pareiorhina rudolphi* and *Pareiorhina brachyrhyncha* was linked to Pleistocene and pre-Pleistocene climatic fluctuations that may have temporarily isolated hillside streams at or near the headwaters of the Ribeirão Grande, producing the events that subsequently led to the sympatry of *Pareiorhina brachyrhyncha* and *Pareiorhina rudolphi*. However, as suggested by Crammer et al. (2008, 2011), Chiachio et al. (2008) and by Roxo et al. (2012a, 2012b), *Pareiorhina brachyrhyncha* and *Pareiorhina rudolphi* do not share an exclusive most recent common ancestor, which negates the hypothesis of Chamon et al. (2005). Additionally, Roxo et al. (2012a) suggested that the origin of the lineages that gave rise to the species of *Pareiorhina* were much older, originating in the Miocene [17.87 (8.24–28.42) million years ago for *Pareiorhina rudolphi* and 6.27 (2.33–11.21) million years ago for *Pareiorhina carrancas* plus *Pareiorhina hyptiorhachis* (*Pareiorhina* sp. 1 in Roxo et al. 2012a)].

## Comparative material

*Isbrueckerichthys alipionis*: LBP 7373, 17, 31.7–81.6 mm SL, municipality of Iporanga, SP, Rio Ribeira de Iguape basin; LBP 2660, 1, 55.1 mm SL, municipality of Iporanga, SP, Rio Ribeira de Iguape basin. *Kronichthys subteres*: LBP 515, 31, 28.4–61.9 mm SL, municipality of Iporanga, SP, Rio Ribeira de Iguape basin. *Neoplecostomus microps*: LBP 8036, 38, 41.3–65.0 mm SL, municipality of Piquete, SP, Rio Paraíba do Sul basin. *Neoplecostomus franciscoensis*: LBP 6489, 50, 42.8–55.9 mm SL, municipality of São Bartolomeu, MG, Rio São Francisco basin. *Neoplecostomus paranensis*: holotype, MZUSP 38572, 71.4 mm SL, municipality of Cajuru, MG, Rio Grande basin. *Pareiorhaphis splendens*: LBP 1117, 20, 32.0–100.0 mm SL, municipality of Morretes, PR, Coastal Drainage. *Pareiorhaphis steindachneri*: LBP 739, 6, 33.8–49.0 mm SL, municipality of Jaraguá do Sul, SC, Coastal Drainage. *Pareiorhina brachyrhyncha*: LBP 12240, 50, 26.4–36.9 mm SL, municipality of Pindamonhangaba, SP, Rio Paraíba do Sul basin. *Pareiorhina carrancas*: LBP 8380, 24, 21.3–38.2 mm SL, municipality of Carrancas, MG, Rio Grande basin. *Pareiorhina cepta*: holotype, MZUSP 111095, 41.5 mm SL, municipality of São Roque de Minas, MG, Rio São Francisco basin, paratypes, LBP 10261, 1, 30.2 mm SL, municipality of São Roque de Minas, MG, Rio Paraíba do Sul basin, LBP 10287, 13, 21.5–43.6 mm SL, municipality of São Roque de Minas, MG, Rio Paraíba do Sul basin, LBP 11835, 19, 25.1–44.0 mm SL, municipality of São Roque de Minas, MG, Rio Paraíba do Sul basin. *Pareiorhina rudolphi*: LBP 8044, 18, 31.7–48.9 mm SL, municipality of Piquete, SP, Rio Paraíba do Sul basin. *Pseudotocinclus juquiae*: LBP1081, 2, 29.0–31.9 mm SL, municipality of Juquitiba, SP, Coastal Drainage. *Pseudotocinclus tietensis*: LBP 2931, 3, 38.6–62.3 mm SL, municipality of Salesópolis, SP, Rio Tietê basin.

## Supplementary Material

XML Treatment for Pareiorhina hyptiorhachis

## Appendix

Editorial Certificate. File format: Portable Document Format File (pdf).
